# Feature-based CBCT self-calibration for arbitrary trajectories

**DOI:** 10.1007/s11548-022-02645-9

**Published:** 2022-05-20

**Authors:** Christian Tönnes, Tom Russ, Lothar R. Schad, Frank G. Zöllner

**Affiliations:** Computer Assisted Clinical Medicine, Mannheim Institute for Intelligent Systems in Medicine, Medical Faculty Mannheim, University Heidelberg, Theodor-Kutzer-Ufer 1, 68159 Mannheim, BW Germany

**Keywords:** CBCT, Calibration, Alignment, Registration, Minimizer

## Abstract

****Purpose**:**

Development of an algorithm to self-calibrate arbitrary CBCT trajectories which can be used to reduce metal artifacts. By using feature detection and matching we want to reduce the amount of parameters for the BFGS optimization and thus reduce the runtime.

****Methods**:**

Each projection is 2D-3D registered on a prior image with AKAZE feature detection and brute force matching. Translational misalignment is calculated directly from the misalignment of feature positions, rotations are aligned using a minimization algorithm that fits a quartic function and determines the minimum of this function.

****Evaluation**:**

We did three experiments to compare how well the algorithm can handle noise on the different degrees of freedom. Our algorithms are compared to Broyden–Fletcher–Goldfarb–Shanno (BFGS) minimizer with Normalized Gradient Information (NGI) objective function, and BFGS with distance between features objective function using SSIM, nRMSE, and the Dice coefficient of segmented metal object.

****Results**:**

Our algorithm (Feature ORiented Calibration for Arbitrary Scan Trajectories with Enhanced Reliability (FORCASTER)) performs on par with the state-of-the-art algorithms (BFGS with NGI objective). nRMSE: FORCASTER = 0.3390, BFGS+NGI = 0.3441; SSIM: FORCASTER = 0.83, BFGS + NGI = 0.79; Dice: FORCASTER = 0.86, BFGS + NGI = 0.87.

****Conclusion**:**

The proposed algorithm can determine the parameters of the projection orientations for arbitrary trajectories with calibration quality comparable to state-of-the-art algorithms, but faster and with higher tolerance to errors in the initially guessed parameters.

## Introduction

Arbitrary trajectories can be used to reduce metal artifacts[1] or cone beam artifacts [2], change field of view [3], and to reduce needed projections [4]. For the quality of these CBCT images, the exact position and rotation at which each projection was acquired is essential. Even though, modern engineering produces machines which can detect their position with a high accuracy, this accuracy is still not sufficient for an artifact-free image. For circular trajectories several algorithms have already been developed [5–7], these algorithms use properties specific to circular trajectories which gives them a significant speed advantage over our proposed algorithm, but it also means they are not usable for arbitrary trajectories. Other calibration methods use phantoms consisting of several metal balls [8–10]. Here the phantom is imaged and then the trajectory can be calibrated using geometric analysis. Only after these two steps the trajectory can be used for the intended image acquisition. This does not work for trajectories that are created on the fly for the current patient and situation, or when the imaging system cannot accurately reproduce the same trajectory. For the calibration of completely arbitrary trajectories only a few papers are published. Ouadah et. al. uses normalized gradient information as the objective function for a Broyden–Fletcher–Goldfarb–Shanno (BFGS) minimization [11]. Chung et al. [12] uses BFGS minimization with an object function based on the distance of Speeded Up Robust Features (SURF) [13] features in simulated forward projections and the acquired images. Both algorithms need multiple hours for a calibration run. Furthermore, both algorithms are evaluated on a regular CBCT image of the same object, which is acceptable for experimental settings, but not for clinical routine examinations. The calibration algorithms has to work with a prior image that is older and differs from the current image.

## Methods

For (arbitrary) trajectories the projections are not dependent on each other, while inter-image consistency conditions exist the projections can also be aligned separately. This leads to a 2D-3D registration for every single projection. Such a registration typically consists of an optimization (also called minimization) algorithm and an objective function. In the approach by Ouadah et. al. [11] or Chung et al. [12] they use the optimization algorithm BFGS to minimize an objective function. This objective function evaluates all projection parameters at the same time and gives an estimate for the correctness, with lower values meaning that the parameters are closer to the correct values. We, instead, propose using different objective functions, one for each parameter. This approach allows us to create objectives, that are sensitive towards change in only one of the parameters.

### Projection and optimization parameters

**Fig. 1:**
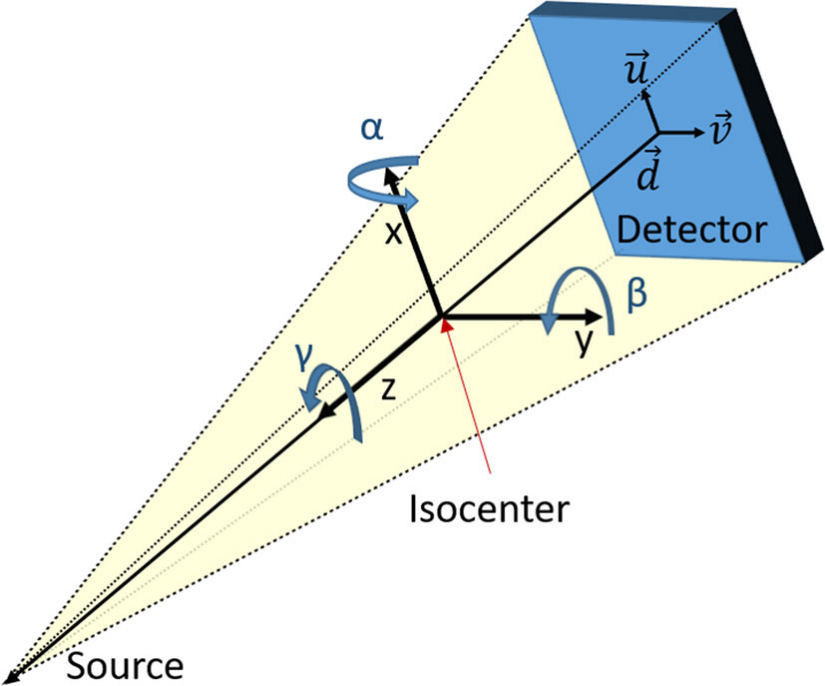
Overview of the coordinate system, parameters and degrees of freedom

In this paper, we use three 3D vectors to describe the position and orientation of a projection (Fig. 1). The vector $\vec {d}$ points to the middle of the detector and $\vec {u}$, $\vec {v}$ contain the direction and distance from the center of one detector element to the center of neighbour elements on the left and top. This definition is equal to the vectors $\vec {d}$, $\vec {u}$ & $\vec {v}$ used by the Astra toolbox [14] to define cone beam geometries.

On these vectors we have the six common degrees of freedom in three dimensional space, that is three translations along the cartesian axes and three rotations around these axes. These optimization parameters use a coordinate system where the *x*- and *y*-axis point in the direction of $\vec {u}$ and $\vec {v}$. The *z*-axis is then given by the direction of the cross product $\vec {u} \times \vec {v}$ and points towards the source. Therefore, the translations and rotations all depend on the current orientation of the projection.

With this coordinate system a movement along the *x*- or *y*-axis corresponds to simple horizontal or vertical shifts of the pixel values in the projection. A rotation around the *x*- or *y*-axis results in points moving horizontally or vertically. Movement in the z-direction zooms the image in or out and rotation around the z-axis rotates the projection without any other change.

### Feature points matching

The algorithmic parts shared by all of our investigated algorithms are feature detection and matching. Features are detected in the real image $p_{ri}$ and a simulated image $p_{si}$ using the Accelerated KAZE (AKAZE) algorithm [15]. This algorithm detects features and computes a descriptor for each feature. Then, the features from one image can be matched to the ones from the other image by comparing the feature descriptors using the hamming distance. The hamming distance is the number of different elements in two vectors of equal length. For every feature in one image we will find the two features in the other image with the lowest hamming distance. These are then used to perform the ratio check described by Lowe et. al. [16] which will discard wrongly matched features. Furthermore, we discard matches if two or more points in one image are matched to the same point in the other image. Also discarded are matches with larger distances than one standard deviation plus the mean distance of all matched points (already excluding multiply matched points). Now we have a set of points $\vec {p_{si}} \in \Omega$ in the simulated images and a function to match them to points $\vec {p_{ri}}$ in the real, acquired, image.

$$\begin{aligned} \vec {p_{ri}}&= \Psi (\vec {p_{si}}) \\ \vec {p_{si}}&= \Psi ^{-1}(\vec {p_{ri}}) \end{aligned}$$

### Correcting shifts

First, we will present the method for correcting shifts along the *x*- and *y*-axis (Listing 1). We calculate the shift along the *x*- or *y*-axis of every pair of points in the real image and the simulated image. For a perfect matching image this shift would be zero. So we simply move the simulated image by the median detected shift.
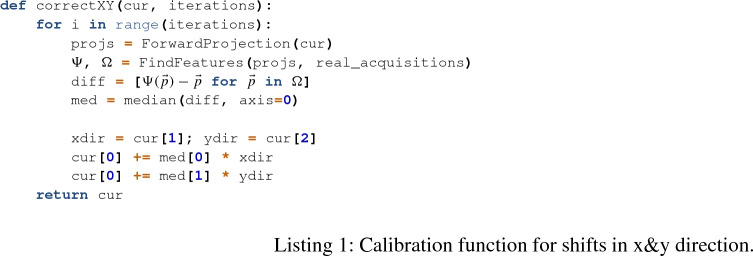


For correcting shifts along the *z*-axis our function calculates the pairwise distance between points within each image and then uses the median ratio of these distances multiplied with the distance between source and iso-center for the shift along the *z*-axis (Listing 2). This ignores misalignment in *x*- or *y*-direction and only considers the magnification. If in both images all distances have the same length the median ratio is one and no zooming is necessary.
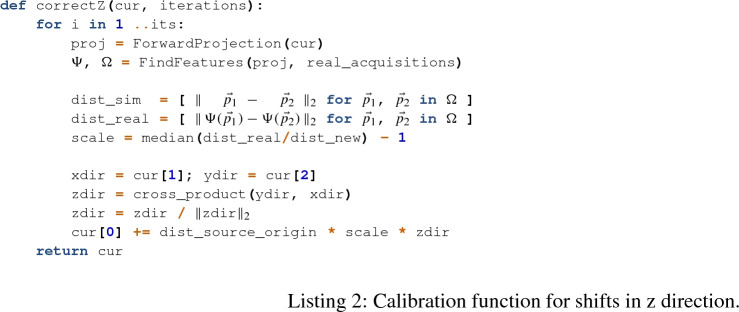


With these functions we can correct the misalignment of the isocenter position. First for the *x*- and *y*-directions then the *z*-direction and another time for *x* and *y* directions. Because we have noisy data and use the median to have less influence from outliers we need multiple calls to both functions.

### Correcting rotations

Secondly, we have to correct the rotations. In contrast to the shift correction we have not found a trivial algorithm, instead we needed an optimizer and a suitable objective function. Despite trying to find objective functions which are specific to each rotation the best results were achieved by measuring the mean euclidean distance between matching feature points.
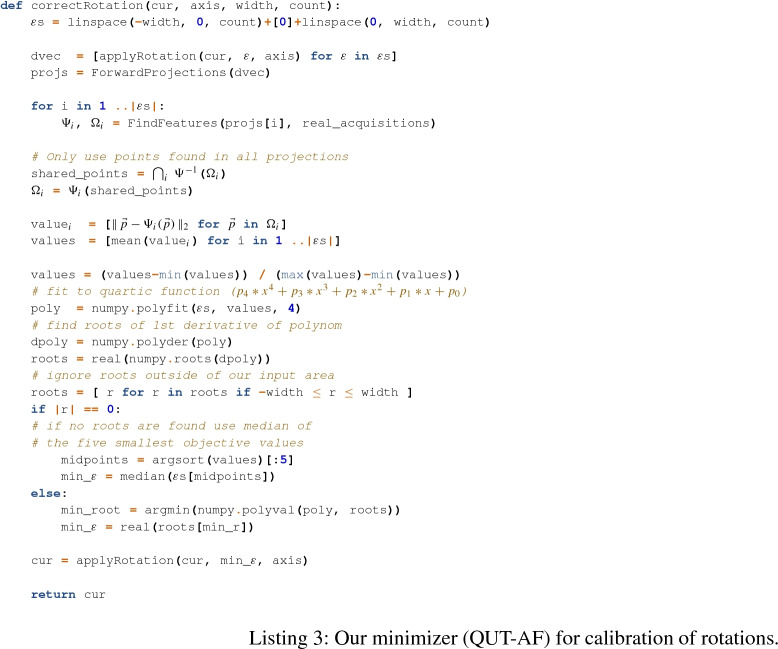


This objective function is too noisy for a simple minimization with an off-the-shelf BFGS optimizer. So, to minimize this objective we developed a simple function. We evaluate the objective at multiple points and then fit a quartic function to these values. The smallest root within the bounds of the used points is the minimized parameter value (Listing 3). We will call this quartic-fitting trajectory alignment function (QUT-AF) during the rest of this paper. A few iterations with decreasing range for the input parameters sufficient for the calibration of the rotational parameters.

For our objective we included another filter for the matched features: Only features present in all images are used. This reduces the noise of the objective function.

### Full algorithm

The two previously described algorithms are interwoven to perform the calibration of all parameters. First we use the functions for correcting shifts, in the order $xy-z-xy$, with three iterations each. Then we use the minimizer for the rotations and between every iteration we do a fast shift correction with only one iteration. After all iterations of the rotation calibration we have a final correction for shift parameters. The full code of our algorithm “Feature ORiented Calibration for Arbitrary Scan Trajectories with Enhanced Reliability” (FORCASTER) is shown in Listing 4.

### Image data

**Fig. 2:**
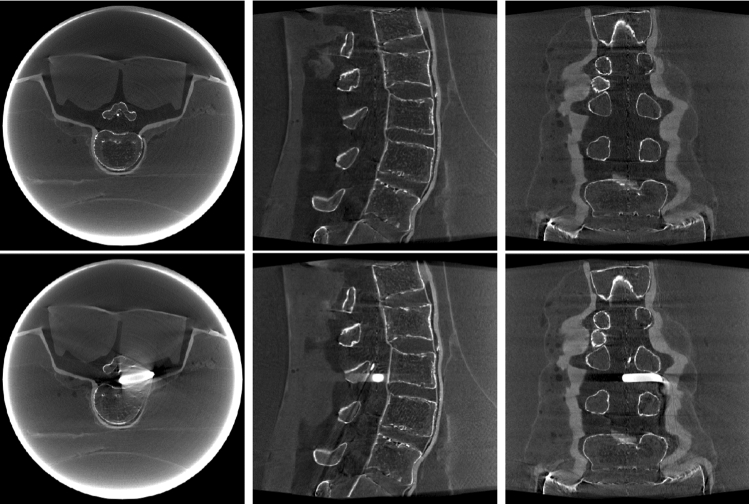
Upper Row: 1st CBCT used as prior. Bottom Row: 2nd CBCT, projections used in calibration

We acquired two CBCT short scans on an Artis Zeego (Siemens Healthineers, Erlangen, Germany) of a lumbar spine phantom. In the first scan a needle was inserted, the second scan had an additional large metal object and the needle position was changed slightly. An axial, saggital and coronal slice of these two images is shown in Fig. 2. We use the first CBCT image, containing only a needle, as our prior image. We will register the projections from the second CBCT scan to this image.

### Evaluation

We evaluated FORCASTER using three experiments and compared it to state-of-the-art algorithms from literature. One of these is the BFGS minimization using the distance between matched feature points [12], the other one is BFGS minimization using the normalized gradient information as the objective function [11]. Additionally, we have two mixed algorithms where we correct the translational errors with our algorithm and then use BFGS with our feature-based objective and NGI for the rotations. We also test a variant of our FORCASTER algorithm using NGI as an objective for the QUT-AF.

For the gradient used by the BFGS algorithm we use a numerical 3-step approximation and run the BFGS multiple times with diminishing step sizes (Table 1). For the algorithms where we mix BFGS optimization with our algorithm for correcting translations we will run a translation correction at the start and after every BFGS run.
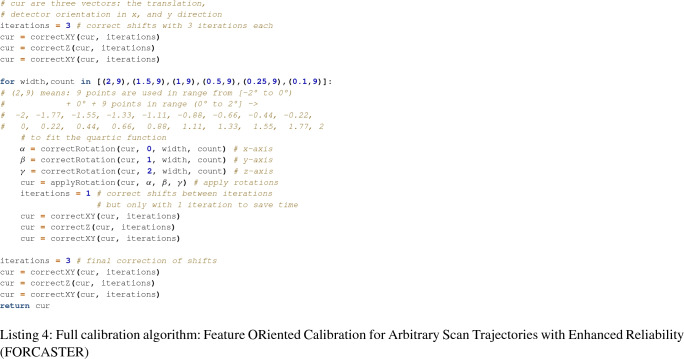

For each BFGS run the stop conditions are an iteration limit of 50 and a gradient norm of less than $10^{-5}$.

#### 1st experiment

The first experiments consist of calibrating a trajectory where only the translational parameters are noisy. To create this trajectory we shifted the initial trajectory in *x*- and *y*-direction by an random amount of pixels from an uniform distribution with the bounds of $-10$ to +10. The zoom factor is chosen from an uniform distribution using the interval from 0.95 to 1. This disturbed trajectory is then calibrated by the different algorithms. The randomization seed is constant, so all algorithms are initialized with the same trajectory. The projections are taken from the second CBCT and the prior image is also the second CBCT.

#### 2nd experiment

In the second experiment we add noise to the rotational and the translational parameters. The noise for rotation parameters are sourced from an uniform distribution of $-2^{\circ }$ to $+2^{\circ }$. Similar to the first experiment all calibration algorithms are initialized with the same noisy trajectory. We use the second CBCT as our prior image and calibrate the projections of the same CBCT.

#### 3rd experiment

Finally, in the third experiment we take the projections from the second CBCT, add noise to all parameters, and then calibrate them using the first CBCT as our prior image.

#### Metrics

We use two metrics for comparison of the results. The first is the structural similarity index (SSIM) [17] evaluated an the projection images. The second is the normalized root mean square error (NRMSE) evaluated on the projections. As a third metric we segment part of the big metal object with simple thresholds and then calculate the Dice-Sørensen-Coefficient. For this we reconstruct the images using the FDK algorithm from the astra toolbox. The segmentation is performed by finding the voxel with the highest value in the rough area where the object should be and then using a threshold at half this maximum value. Afterwards a morhpological opening with a $3\times 3\times 3$ kernel and a connected component analysis is used to get the segmentation.

#### System specification

Our algorithms were run on a system with an Intel Core i7-4790K, 32 GB RAM, NVIDIA GeForce RTX 2070 SUPER. Due to each projection being independent from the others, all these algorithms can be easily parallelized. We use as many parallel processes as the CPU has logical cores, so 8 for the i7. We use python 3.7.6 and the packages: astra-toolbox 1.9.9.dev [14], scipy 1.6.1, skimage 0.18.3, numpy 1.17.4, opencv 4.5.1-dev.

## Results

### 1st experiment

The results from the first experiment, where we only had translational noise to calibrate, are in Table 2. We have seen in this experiment, that after three iterations of each correction step no further corrections are made. So whenever we mention our translation correction algorithm it will be three iterations of *x*, *y* correction, three times *z* correction and three times *x*, *y* correction. The BFGS optimizer with NGI optimizer performed very poorly in all metrics. We therefore added another run where the translational noise is reduced by halve to a pixel shift of $-5$ to $+5$.

**Table 1:** Step sizes for the gradient approximations

	Parameter	1st run	2nd run	3rd run*
Our objective	Rotations	$0.25^{\circ }$	$0.025^{\circ }$	
	Translations	2	1	
NGI objective	Rotations	$0.25^{\circ }$	$0.05^{\circ }$	$0.01^{\circ }$
	Translations	3	2	1

*Only for NGI objective

**Table 2:** Results for the 1st experiment

Algorithm	SSIM	NRMSE	Dice	Runtime [hh:mm]
Our algorithm	1.00	0.0017	1.00	00:16
BFGS (Our objective)	0.96	0.0287	0.99	05:30
BFGS (NGI objective)	0.71	0.2456	0.82	07:20
BFGS (NGI objective)*	0.91	0.0794	0.97	05:13

*Reduced translational noise

**Table 3:** Results for the 2nd experiment

Algorithm	SSIM	NRMSE	Dice	Runtime [hh:mm]
FORCASTER	0.97	0.0237	0.99	03:14
FORCASTER (NGI Objective)	0.96	0.0322	0.99	01:16
Mixed BFGS (Our Objective)	0.91	0.0559	0.98	06:47
Mixed BFGS (NGI Objective)	0.98	0.0224	0.99	02:47
BFGS (Our Objective)	0.89	0.0606	0.97	13:01
BFGS (NGI Objective)	0.67	0.2491	0.94	11:32
BFGS (NGI Objective)*	0.90	0.0644	0.96	13:43

*Reduced translational noise

**Table 4:** Results for the 3rd experiment and the difference of the prior image to the calibrated image

Algorithm	SSIM	NRMSE	Dice	Runtime [hh:mm]	Iterations**
Prior difference	0.86	0.3380	0.88	Ø	
FORCASTER	0.83	0.3390	0.86	02:59	9
FORCASTER (NGI objective)	0.84	0.3390	0.87	01:03	9
Mixed BFGS (Our objective)	0.79	0.3406	0.87	09:21	24
Mixed BFGS (NGI objective)	0.85	0.3387	0.88	03:47	120
BFGS (Our objective)	0.79	0.3410	0.87	20:54	31
BFGS (NGI objective)	0.63	0.3743	0.82	10:23	124
BFGS (NGI objective)*	0.79	0.3441	0.87	10:36	69

*Reduced translational noise; **Iterations of FORCASTER and BFGS not comparable due to different minimzing algorithms

### 2nd experiment

In Table 3 are the results for the second experiment. Here we can see that our minimizer performs equally good with our objective and the NGI objective. Furthermore we can see in all three metrics, that our minimizer performs better than the algorithms based on the BFGS optimizer.

**Fig. 3:**
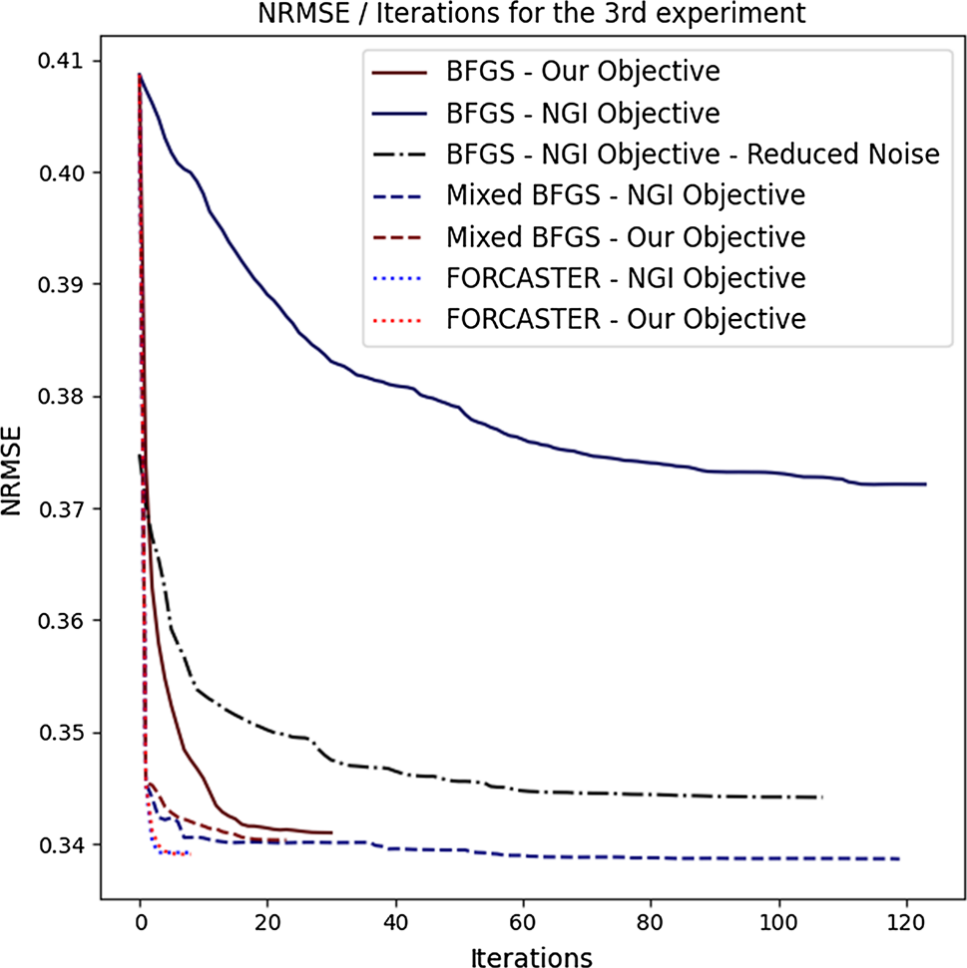
The NRMSE plotted over the iterations for the algorithms and data used in the 3rd experiment

**Fig. 4:**
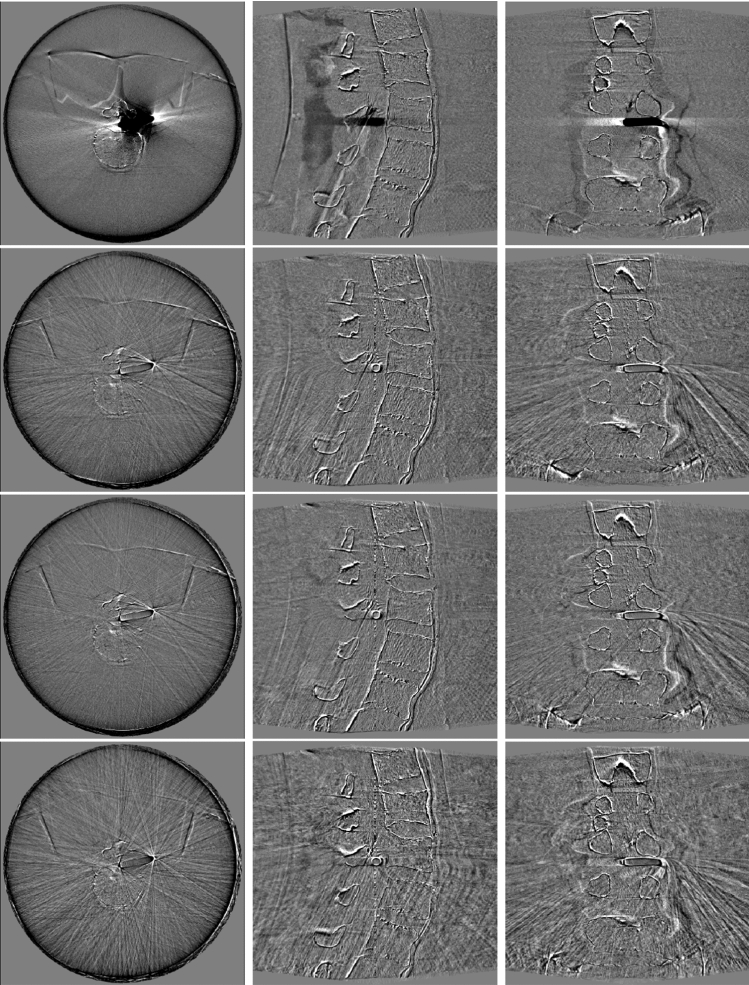
Error between reconstructions from Experiment 3 and the actual image. First row: error of the prior image. Second row: error of FORCASTER. Third row: error of mixed BFGS with NGI. Bottom row: error of BFGS with NGI (reduced translational noise)

**Fig. 5:**
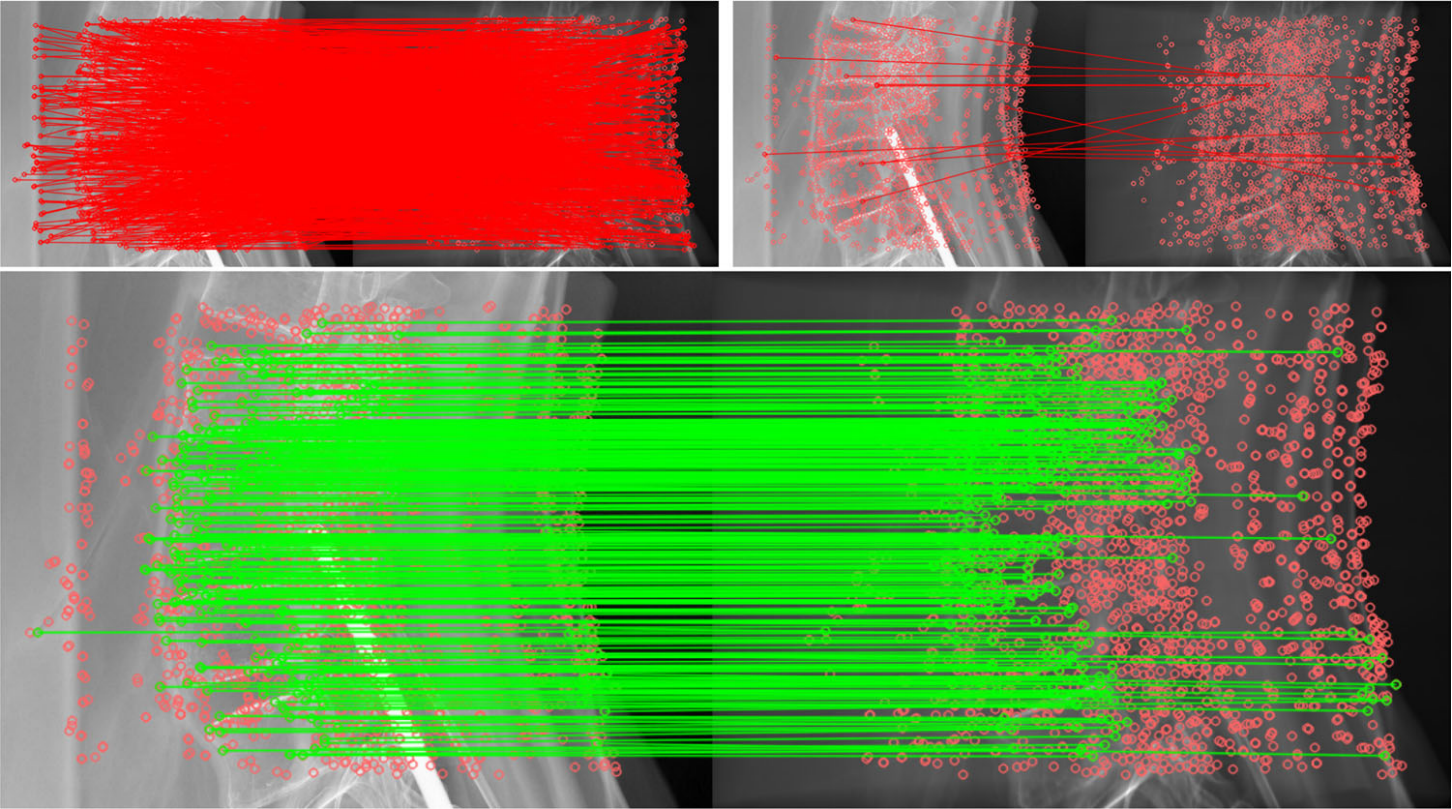
Top Left: Matches discarded by Lowe’s ratio check. Top Right: Matches discarded by double match and outlier filter. Bottom: Remaining matches after filtering

### 3rd experiment

In the third experiment the images were calibrated on the first CBCT and compared to the second CBCT, therefore all metrics (Table 4) are slightly worse than those of the second experiment. Still, it shows similar results. Our algorithm is on par with the one from literature. BFGS using our feature distance objective performs worse than the NGI objective for calibrating the rotations but better in relation to the translations. In Fig. 4 are images of the reconstructions for the calibration done by our minimizer and the Mixed BFGS with NGI objective. The selected slices are the same as in Fig. 2.

The runtime for the full BFGS algorithms are, as expected, very high. The NGI objective always performed much faster than our objective unrelated to the underlaying optimizer. The fastest algorithm was the one using our translation correction using feature matching and the QUT-AF minimizer with the NGI objective. This algorithm needed only 10% of the time the state-of-the-art algorithm of BFGS+NGI took. In Fig. 3 the NRMSE is plotted over the iterations. The mixed and FORCASTER algorithms all start with the same steps, therefore they have the same steep decline at the first iteration. The total number of iterations for every algorithm can also be seen in Table 4. It shows, that the total number of iterations is only slightly decreased when moving the translation correction out of the BFGS minimization.

## Discussion

We have shown, that FORCASTER can achieve an accuracy that is comparable to the state of the art for the problem of arbitrary task-based trajectory calibration. Even if we use a prior image, that has significant changes in contrast to the projections, we can successfully calibrate the trajectory. We deem this to be an important property for online calibration algorithms if task-based trajectories should be integrated into clinical practice.

Furthermore, our results show that it is possible to separate the optimization of rotations and translations without an impact to the calibration performance. The mixed BFGS algorithms had a slightly lower NRMSE than the full BFGS algorithms. The FORCASTER algorithm, going one step further and optimizing the parameters serially, but with six loops, also achieved a lower NRMSE than a full BFGS.

One obvious problem with feature matching are wrong matches. Most mismatched features are eliminated by Lowe’s ratio check. From the remaining matches  5% are still incorrect. These are removed by the outlier and double match filters. In Fig. 5 the matches discarded by the filtering steps and the remaining matches are displayed for one projection.

Using the distance between features for the objective function given to a BFGS minimizer gave poor results when calibrating rotations. This is probably due to the noisiness of this objective. Our approach with QUT-AF of fitting a quartic function is more robust than the 2-point or 3-point numerical derivative used for BFGS, but calculating this derivative with more points increases the computational cost and thus further slows down the minimization.

Even though our algorithm is, with a runtime of 3 hours, faster than a BFGS minimization (10 h), its long runtime is still a problem that needs to be solved. One approach could be by leveraging the pair-wise independence of each projection and use more parallel processing. This could be done on a high-performance cluster or on a GPU with enough memory. Alternatively, developing a faster objective function for the calibration of the rotations might improve processing speed. Here our results show that the mixed approach of a feature-based objective for translations and the NGI objective for rotations is three times faster than our algorithm.

A way to estimate the needed rotation from two simulated projections, similar to how we estimated the needed translation, would speed up the calibration immensely. Removing the translational minimization from the BFGS optimizer saved more than 10 h of computation. Here the matched features give a plethora of information on what changes between slightly rotated projections which is hopefully enough for a simple and fast algorithm.

Also the feature-based approach showed a high tolerance to wrongly guessed start parameters. An adaption to further remove the need for an accurate initial guess would help with images that do not have attached positions. This is the case for continuous acquisition on an Artis Zeego System. Only the starting position is exported to the DICOM but not the positions of all subsequent frames. In conclusion we have presented a viable approach to calibration which uses techniques that are in this context not well explored but are interesting for further research.

In conclusion we have shown, that feature-based calibration is a contender to the state of the art calibration algorithms. With equal quality, but shorter runtime and higher robustness to wrong start parameters.

## Supplementary information

If your article has accompanying supplementary file/s please state so here.

Please refer to Journal-level guidance for any specific requirements.
